# The Influence of Preoperative Physical Activity on Postoperative Outcomes of Knee and Hip Arthroplasty Surgery in the Elderly: A Systematic Review

**DOI:** 10.3390/jcm9040969

**Published:** 2020-03-31

**Authors:** Sebastiano Vasta, Rocco Papalia, Guglielmo Torre, Ferruccio Vorini, Giuseppe Papalia, Biagio Zampogna, Chiara Fossati, Marco Bravi, Stefano Campi, Vincenzo Denaro

**Affiliations:** 1 Department of Orthopaedic and Trauma Surgery, Campus Bio-Medico University of Rome, 00128 Rome, Italy; s.vasta@unicampus.it (S.V.); r.papalia@unicampus.it (R.P.); f.vorini@unicampus.it (F.V.); g.papalia@unicampus.it (G.P.); b.zampogna@unicampus.it (B.Z.); s.campi@unicampus.it (S.C.); denaro@unicampus.it (V.D.); 2 Department of Movement, Human and Health Sciences, University of Rome “Foro Italico”, 00100 Rome, Italy; chiara.fossati@uniroma4.it; 3 Department of Physical Medicine and Rehabilitation, Campus Bio-Medico University of Rome, 00128 Rome, Italy; m.bravi@unicampus.it

**Keywords:** knee, hip, arthroplasty, physical activity, elderly, prehabilitation

## Abstract

Total hip arthroplasty (THA) and total knee arthroplasty (TKA) represent two of the most common procedures in orthopedic surgery. The growing need to avoid physical impairment in elderly patients undergoing this kind of surgery puts the focus on the possibility to undertake a preoperative physical activity program to improve their fit and physical health at the time of surgery. A systematic review has been carried out with online databases including PubMed-Medline, Cochrane Central and Google Scholar. The aim was to retrieve available evidence concerning preoperative physical activity and exercise, before total knee or total hip arthroplasty in patients older than 65 years, and to clarify the role of this practice in improving postoperative outcomes. Results of the present systematic analysis showed that, for TKA, most of the studies demonstrated a comparable trend of postoperative improvement of Visual Analogue Scale (VAS), range of movement (ROM) and functional scores, and those of quality of life. There is insufficient evidence in the literature to draw final conclusions on the topic. Prehabilitation for patients undergoing TKA leads to shorter length of stay but not to an enhanced postoperative recovery. Concerning THA, although currently available data showed better outcomes in patients who underwent prehabilitation programs, there is a lack of robust evidence with appropriate methodology.

## 1. Introduction

The prevalence of osteoarthritis in the elderly population is relevant, especially for lower limb weight-bearing joints. Total knee arthroplasty (TKA) and total hip arthroplasty (THA) are the two major surgeries for end-stage osteoarthrosis, usually advocated when all conservative treatments are inefficient. The healthcare-related economic burden is extensive for these surgeries, especially concerning postoperative hospitalization, leading in recent years to the development of several fast-track strategies aimed to improve results and decrease hospital stay expenses [1,2]. 

Arthroplasty aims to restore the function of the joint and soothe the pain derived from bone-on-bone arthritic conditions. After hip and knee arthroplasty, a consistent rehabilitation program is usually undertaken in order to provide the patient with the adequate strength and mobility to bear the prosthetic implant and to guarantee a correct function [1]. Isometric strengthening of the muscle responsible for the index joint movement (gluteal muscles for the hip and quadriceps for the knee) is a key feature of postoperative rehabilitation, providing the limb with the appropriate muscular support for the mobilization of the hip or the knee. Furthermore, an antagonist stretching program is mandatory to achieve a full range of movement (ROM), avoiding postoperative stiffness and walking disabilities. In a consecutive phase, neuromuscular education to walking is advocated to eventually restore the locomotor function of the limb [3]. 

In recent years there has been growing interest in the possibility to prepare patients for surgery through a “prehabilitation” program, composed of strengthening and stretching exercises in the immediate preoperative period. The focus of several research projects at present is to understand whether a prehabilitation program of specific exercises or physical activities may influence and improve the postoperative outcomes of the patients. Physical exercise has been already reported to be beneficial in the knee and hip OA as a conservative treatment [4]. Activity improves function and decreases pain; thus, it is actually suggested for those patients affected, independently from the schedule of the surgery. However, it is not clear if specific exercise programs improve surgical outcomes and postoperative parameters of the patient, including the length of stay in the hospital and the quality of life. The aim of the present systematic review of the literature is to collect evidence concerning preoperative programs of activity and exercises for those patients scheduled for hip and knee arthroplasty. A specific focus of our research frame is the elderly, as there are major concerns about functional recovery and length of hospital stay in this sub-population. This intension was firstly determined from the examination of previous literature, where heterogeneity of population characteristics compromised the internal consistency of results of systematic reviews and meta-analyses [5]. The primary endpoint is to clarify the impact of specific training on subjective and objective surgical outcomes; the secondary endpoint focuses on the influence on postoperative parameters including the length of hospitalization and the quality of life of the patient. 

## 2. Methods

The present systematic review was carried out in accordance with the Preferred Reporting Items for Systematic Review and Meta-analysis (PRISMA) guidelines and followed the Cochrane methodology for systematic reviews [6]. However, no protocol for systematic review has been registered. Furthermore, PICO (Poipulation, Intervention, Comparison, Outcome) methodology has been used to formulate the study hypothesis. According to PICO, the following elements have been used to frame the study question:

Population—patients who are candidates for TKA or THA;
Intervention—prehabilitation; preoperative physical activity program;
Comparison—no preoperative intervention;
Outcome of interest—postoperative functional outcomes and length of stay.

### 2.1. Criteria for Considering Studies for This Review

The studies considered for inclusion were randomized controlled trials (RCT), prospective cohort studies (PCS), case-control studies (CCS) and case series (CS). Case reports, reviews and meta-analyses were excluded. Furthermore, basic science and in-vitro studies, biomechanical and cadaver evaluations were excluded. Studies considered should concern the preoperative physical activity in elderly patients that were scheduled for TKA or THA. According to WHO’s definition of the elderly, only studies where the average age of the cohorts was greater than 65 years were considered.

### 2.2. Primary Outcomes

Subjective and objective clinical measurements were considered as the primary outcome of the analysis, taking into account the clinical scores reported, which included Knee injury and Osteoarthritis Outcome Score (KOOS) Western Ontario McMaster University Osteoarthritis Index (WOMAC), Knee Society Score (KSS) and range of motion (ROM) of the index joint. Furthermore, the outcomes of physical performance tests were considered, including the 6 min walking test (6-MWT), the time up and go test (TUG), and gait speed and distance.

### 2.3. Secondary Outcomes

Postoperative length of stay was the main secondary outcome considered. Furthermore, the quality of life of the patients after surgery was considered, measured through Short Form-36 (SF-36) and the quality of life section of the Knee injuries and Osteoarthritis Outcome Score (KOOS).

### 2.4. Search Methods for Identification of Studies

Online databases were searched for relevant articles, including PubMed-Medline, Cochrane Central and Google Scholar. The search was carried out between March and November 2019. Search strings used were the following: (“exercise” [MeSH Terms] OR “exercise” [All Fields] OR (“physical” [All Fields] AND “activity” [All Fields]) OR “physical activity” [All Fields]) AND (“aged” [MeSH Terms] OR “aged” [All Fields] OR “elderly” [All Fields]) AND (“arthroplasty” [MeSH Terms] OR “arthroplasty” [All Fields]); Prehabilitation [All Fields] AND (“aged” [MeSH Terms] OR “aged” [All Fields] OR “elderly” [All Fields]) AND (“arthroplasty” [MeSH Terms] OR “arthroplasty” [All Fields]).

**Figure 1:**
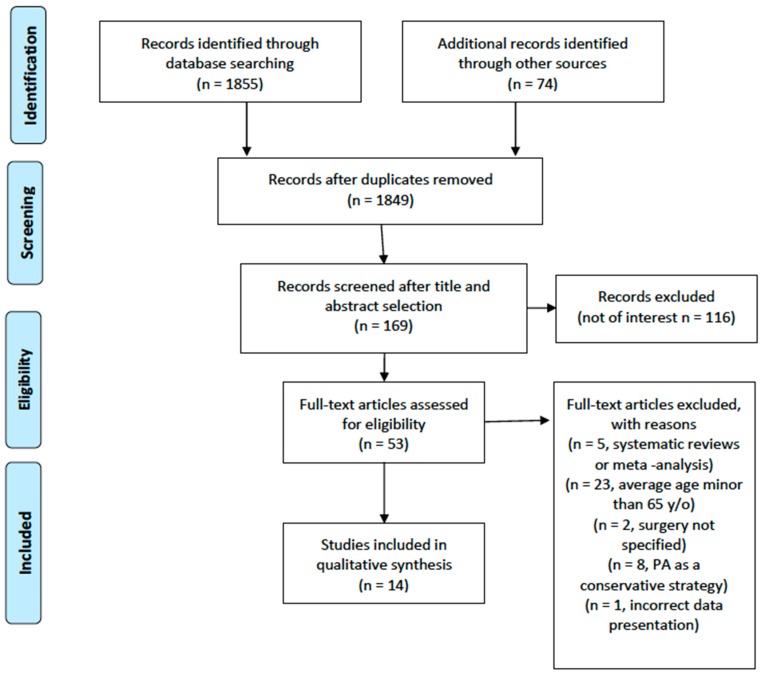
Preferred Reporting Items for Systematic Review and Meta-analysis (PRISMA) flow diagram. PA: Physical Activity

No time interval was set for publication date. The studies retrieved were firstly screened by title, and where relevant, the whole abstract was read. After a first selection and exclusion of not-relevant papers, the full text of the potentially eligible articles was retrieved and read by two reviewers for eventual inclusion. Discordant opinions were solved through the consultation of a third reviewer. After the electronic search was completed, the bibliography of the relevant articles included was screened manually to identify further papers potentially missed in the electronic search. The search process is summarized in the flow diagram in Figure 1.

### 2.5. Data Collection and Analysis

Data were extracted from the included articles according to the primary and secondary outcomes considered for the aim of this review. After extraction, generic data concerning the paper and specific outcome data were reported in tables. The protocol of preoperative physical activity intervention was analyzed and reported in a specific table. For an appropriate presentation of data, the results were divided on the basis of the surgery (TKA or THA).

### 2.6. Risk of Bias Assessment

Given the heterogeneity of the included studies, two different critical appraisal tools were utilized. For randomized clinical trials, the Cochrane risk of bias assessment tool was used, providing a grade of risk (low or high risk) of bias for the index study in five elements of the study design (sequence generation, allocation concealment, blinding, incomplete data addressing and selective reporting). For non-randomized studies, the Methodological Index for Non-Randomized Studies (MINORS) score was used.

## 3. Results

### 3.1. Results of the Search

From the electronic search, a total of 1855 articles were retrieved. One of the authors (G.T.) screened the results by title and abstract and manually searched the bibliographies of the relevant papers, especially reviews and meta-analyses. Of the articles retrieved through electronic and manual search, 14 were finally included (Figure 1) [7,8,9,10,11,12,13,14,15,16,17,18,19,20].

### 3.2. Included Studies

Of the included studies, 12 were RCT of Level Of Evidence (LOE) I, 1 was a prospective case–control study of LOA II, and 1 was a CS of LOE IV [7]. Of these, 10 presented outcomes of TKA [7,11,12,13,14,15,16,17,18,19], 3 of THA and one evaluated both TKA and THA results [8,9,10].

### 3.3. Excluded Studies

Several studies retrieved were excluded for the following main reasons: average age of the cohort < 65 years [20,21], reviews or meta-analyses [22] and/or no surgery scheduled (assessment of physical activity as a conservative treatment).

### 3.4. Demographic Data

The included studies reported data on a total of 1175 patients, with an average age ranging from 66 to 76.9 years. A total of 1096 patients were scheduled for TKA, while 79 were scheduled for THA. Details on demographic data of the cohorts are shown in Table 1 and Table 2.

### 3.5. Total Knee Arthroplasty

#### 3.5.1. Main intervention

Studies concerning knee arthroplasty focused on preoperative muscle strengthening or proprioceptive exercises. Timing of intervention ranged between 2 and 12 weeks before surgery. Specific training included strengthening with elastic resistance band [19], combined land-based and pool-based exercises [18], supervised proprioceptive training [17] and progressive resistance training [12,16]. Several papers intervened by instructing patients of the study group in a home-based exercise program [14,15], while several other administered supervised training sessions [13,16,17,18,19]. Some of the studies reported a continuation of the activity program in weeks 4 to 8, after surgery [16,19]. In Table 3 specific programs were summarized in comparison to control group activity.

#### 3.5.2. Clinical Outcome Data

Preoperative strengthening showed a positive effect on perioperative outcomes in several of the included studies. In the study by Evgeniadis et al., SF-36 score was better for the study group, in the immediate preoperative setting, though this difference was not significant [19]. Similarly, no significant difference occurred in SF-36 in the paper by Gill et al., as well as the functional outcomes, assessed through WOMAC score [18]. A progressive resistance training program, administered in the study by Skoffer et al. [16], showed improved functional recovery in the patients of the study group assessed through TUG test, 30 s chair stand test (30sCST) and determination of knee flexion strength; however, there was no difference in KOOS, VAS and on a 100-point quality of life rating scale. Similarly, no difference was observed in WOMAC score after a preoperative proprioceptive training in another recent study [17]. Contrary to these results, the progressive strength training program advocated in the study by VanLeeuwen et al. yielded comparable functional results between groups in terms of 6MWT and chair stand [12]. Similarly, in the paper by Aytekin et al. [20], KOOS and VAS were comparable between intervention group (home-based strengthening and stretching exercises) and controls. In the case series by Twiggs et al. [7], a significant correlation was found between preoperative step count and KOOS (activity of daily living subscore) immediately before surgery (rho = 0.282, *p* < 0.05). A trial reported that the ROM and the Iowa Level of Assistance Scale (ILAS) were significantly better in the study group after the completion of the supervised program, 8 weeks after surgery [19]. Conversely, the paper by Matassi et al. [14] reported a better recovery of knee ROM after a preoperative home-based general strengthening program. Nevertheless, a study reported no difference among groups in ROM and VAS in the early postoperative days after a pre-surgery home-based strengthening program [15]. Similarly, no difference in ROM was observed in the trial by Beaoupre et al. [11]. Hospital length of stay was assessed in five papers. The article by Huang et al. [15] reported a significantly lower medical expenditure for the patients that participated in the home-based preoperative program (7 ± 2 vs. 8 ± 1 days; *p* = 0.001). A shorter hospital stay was also reported by Matassi et al. (9.1 ± 2.1 vs. 9.9 ± 2.3 days) [14] and in the study by Williamson et al. for those patients that underwent home-based [14] or supervised (6.5 vs. 7.7 days) [13] strengthening programs. The major difference registered, though not statistically significant, occurred in the study by Aytekin et al., where the average length of stay was longer in the intervention group (5.5 ± 2 vs. 7.9 ± 2.3 days; *p* > 0.05) [20]. A prospective case series by Twiggs et al. [7] showed a poor positive correlation (rho = −0.114, *p* > 0.05) between preoperative step count and hospital length of stay.

#### 3.5.3. Methodological Evaluation

Nine of the included studies concerning TKA were level I RCT, and one was a level IV CS. The methodological assessment was carried out through Cochrane Risk of Bias Assessment Tool for the nine trials and the MINOR score for the non-comparative series. According to the evaluation, the studies all had some major flaws in methodology, except for one [16], where only blinding bias was considered high. Among trials, the evaluation showed that major limitations were observed concerning the blinding bias because blinding of physical activity is actually impossible to achieve. Furthermore, several studies did not describe any statistical method for addressing incomplete data, thus a high risk of bias in this field was also reported. Selective reporting was also a bias of several studies, which focused only on one or a few aspects of postoperative recovery.

### 3.6. Total Hip Arthroplasty

#### 3.6.1. Main intervention

Considering the studies reporting outcomes of THA, three papers [8,9,10] showed outcomes of patients that underwent a program of muscular strengthening. Specifically, a personalized activity divided into progressive phases [8] was administered, either home-based or supervised [9,10]. The timing of the activity program ranged between 6 and 3 weeks before surgery, with a schedule of two sessions per week. In Table 3 specific programs were summarized in comparison to control group activity.

#### 3.6.2. Clinical Outcome Data

Although in one trial no difference occurred between study and control groups [8], the other two papers [9,10] reported better functional outcomes in the intervention group. Specifically, the time up and go (TUG) test and 6-minute walking test (6MWT) were better performed at 6 weeks after surgery by those patients managed with preoperative program of strengthening [9]. Similarly, 6MWT was improved in the study group at 12 and 24 weeks, and a greater stride length and increased gait speed were observed at 3, 12 and 24 weeks after surgery in those patients treated with a personalized activity program [9]. In the study by Hoogeboom et al., no difference was observed in length of stay, with an average time of 6 days in the study group (range 5–22 days) and of 6 days in the control group (range 4–7 days), with a *p* = 0.228 [8].

#### 3.6.3. Methodological Evaluation

All the included studies concerning THA were level I RCT. The methodological assessment was carried out through Cochrane Risk of Bias Assessment Tool (Table 4). According to the evaluation, the three studies had one or more major flaws in methodology; therefore, the risk of bias within a single trial was high for the three studies. Among trials, evaluation also showed some major limitations, especially concerning allocation concealment, which was at high risk of bias. Furthermore, selective reporting was at high risk of bias for two studies [9,10] where only functional outcomes were reported, and no data concerning length of stay and perioperative outcomes were shown.

## 4. Discussion

The primary endpoint of the present investigation was to assess whether a preoperative activity program impacts on the functional recovery. Concerning TKA, discordant results were reported concerning functional assessment with 6MWT and TUG, either considering studies reporting similar protocols of preoperative exercise [12,16]. However, most of the studies demonstrated a comparable trend of postoperative improvement of VAS, ROM and functional scores (KOOS and WOMAC) and those of quality of life (SF-36). Conversely, clear evidence can be observed concerning the postoperative length of stay, as all the studies analyzing length of stay demonstrated a shorter length of stay in those patients undergoing the preoperative activity program. Regarding the studies on THA, stronger evidence is available on the positive influence of preoperative activity on functional recovery [9,10]. Conversely, length of stay was comparable between groups, although only one study reported these data. As in previous review works, these results do not achieve clinical relevance, although most are statistically significant [23,24].

The preoperative exercise programs were significantly variable and differed especially for the type of exercise, while the duration was similar, as almost all the studies reported a protocol of activity within the 6 weeks preceding the index surgery. The most relevant point concerning the activity program was supervision of the exercises by a trainer or a physical therapist. Some of the studies advocated a home-based program [9,14,15], while several other administered supervised training sessions [8,13,16,17,18,19,20]. A slight modification of the exercise program did not yield significant differences in results, in fact in the trial by VanLeeuwen et al. [12] no difference occurred between groups where progressive strength training was added to standard muscle strengthening. Conversely, progressive resistance training compared to daily life activities led to significantly better functional results [16]. These differences in study protocols made it difficult to compare results among the trials and prevented the authors to carry out a meta-analysis of the reported data. Although the conclusion of the single trials is often clear and the evidence seems to be defined, the summary of the results cannot be considered conclusive given the inhomogeneity of the study protocols.

Strength of the quadriceps muscle is one of the most important contributors to functioning of the knee, specifically in people with knee OA [22,25]. However, currently investigated studies failed to show the effectiveness of preoperative strengthening programs in enhancing postoperative recovery after TKA. The American College of Sports Medicine guidelines reported that to improve strength, muscle mass and endurance, exercises with a resistance of ~ 60% to 80% of the individual’s 1 repetition maximum and titratable progression are required [26]. Moreover, a previous study showed that an 8-week period of exercise is necessary to produce significant improvements in pain and function and in objective measurements of muscle functioning in OA patients [27]. All the studies analyzed in the present review reported an intervention length ranging between 2 and 6 weeks, which is shorter than the minimum length, and most of them used home-based exercise programs, so it is difficult to have trustable data on whether the patients followed the indications for resistance thresholds or not. Those two factors may be responsible for not obtaining significantly better outcomes in patients who underwent prehabilitation compared to those who did not.

Concerning methodology, almost all the papers included had several biases assessed through the Cochrane Risk of Bias Assessment Tool. This evaluation highlighted that most of the trials included had several methodological flaws, especially concerning allocation concealment and blinding of the participants. However, it is relevant to understand that for active exercise programs blinding is actually impossible. Apart from this incongruity, the trials were designed in an appropriate manner and always included a control group for which normal daily activities were advised in the time before surgery. In addition, other relevant biases that affect the quality of the included trials are the selective report and the small cohorts of included patients. Especially the paucity of the cohort affects significantly the final result of a study, and the power analysis was not reported in most of the included papers. This may lead to an unpredictable overestimation or underestimation of the results. Furthermore, lack of appropriate power prevents the reader to truly understand and weigh the importance of the presented data in view of a clinical application of the evidence. Selective reporting was also a source of frequent bias, with many studies reporting data on functional outcomes (including WOMAC, KOOS) without reporting information about quality of daily life (SF-12 or SF-36) or vice-versa. This does not allow a thorough evaluation of the patients. All of these biases should be taken into account to carry out novel studies on this topic.

Potential limitations of the present systematic review include the narrow electronic research frame, as only two online databases have been searched. Furthermore, given the language capabilities of the author, only studies published in English have been retrieved and analyzed. This is a bias which may have reduced the pool of retrieved papers. However, the main strength of this study is the systematic methodology, strictly adhering to PRISMA guidelines and PICO process for formulation of the research question. Furthermore, as an added value in comparison to previous similar works, our study strictly focuses on elderly patients, for which outcomes seem to be more homogeneous and consistent than those in other age groups. This is especially true for studies concerning total knee arthroplasty.

## 5. Conclusions

Although there are insufficient data to draw definitive conclusions, prehabilitation for patients undergoing total knee arthroplasty leads to a shorter length of stay, but not to an enhanced postoperative recovery. Similarly, concerning total hip arthroplasty, although currently available data showed significantly better outcomes in patients who underwent prehabilitation programs, there is a lack of robust evidence in its favor. Although the presented results do not achieve appropriate clinical relevance, it is useful to know that in small cohorts preoperative physical training shortens the length of stay in the hospital, which is a remarkable result in an era of increased attention to healthcare expenses. Thus, considering prehabilitation measures are non-invasive and low-cost activities, it would be worthwhile to suggest them to patients undergoing total joint arthroplasty.

## Figures and Tables

**Table 1:** TKA.

Study	Type of Study	LOE	Number of Patients	Mean Age (y)	Type of Scheduled Surgery	Type of Intervention	Outcomes Summary
Evgeniadis et al., 2008	RCT	I	53	68.76	TKA	General strengthening exercise program 3 weeks before surgery vs. specific strengthening exercise program 8 weeks postoperatively	Preoperative SF-36 was slightly better in patients treated with preoperative strengthening. ILAS and active ROM resulted significantly improved at the termination of the program in the group treated with strengthening postoperatively
Gill et al., 2009	RCT	I	82	70.3	TKA, THA	Land-based vs. pool-based preoperative exercise programs, 6 preoperative weeks	Significant improvement in postoperative performance for both groups, although no difference occurred between groups in terms of WOMAC and SF-36
Gstoettner et al., 2011	RCT	I	38	72.8 (study group) 66.9 (control group)	TKA	Preoperative proprioceptive training	Stance stability improved significantly in the study group at 6 weeks after surgery (Biodex Stability System evaluation). No difference between study and control group occurred in postoperative (6 weeks) WOMAC and KSS
Huang et al., 2012	RCT	I	273	70	TKA	Home-based rehabilitation educational program (4 weeks before surgery)	Medical expenditure of hospital stay in the study group was significantly lower (*P* = 0.001). VAS and ROM was not significantly different in patients of both groups in the early day after surgery (5 days after admission)
Matassi et al., 2012	RCT	I	122	66 (study group), 67 (control group)	TKA	Home-based exercise program (6 weeks before surgery)	Exercise program improved the recovery of knee motion and yielded a shorter hospital stay. Differences were balanced in the long-term follow-up.
Skoffer et al., 2015	RCT	I	59	70.7 (study group), 70.1 (control group)	TKA	Progressive resistance training in the 4 preoperative weeks	Significant differences were found in the study group when compared to controls in terms of 30sCST, TUG, knee flexors strength. No difference was found in KOOS, VAS and a 100-points quality of life rating scale.
Twiggs et al., 2017	CS	IV	91	67.5	TKA	Fitbit wristband activity goals (step count)	Poor positive correlation (not statistically significant) between higher preoperative step count and hospital stay. KOOS QOL was significantly associated to step count 6-weeks postoperatively and KOOS Pain was significantly correlated to step count at preoperative and postoperative day 2–4 step count.
VanLeeuwen et al., 2014	RCT	I	22		TKA	Standard training with additional program of progressive strength training	No difference was found between groups in 6MWT and chair stand. Moreover, no difference was found in recovery time
Williamson et al., 2007	RCT	I	181	72.4 (Acupuncture group) 70 (Physiotherapy group) 69.6 (Controls)	TKA	Supervised strengthening exercises 6 weeks before surgery	Shorter in-patient stay was observed in the physiotherapy group (1 day less than other groups)
Aytekin et al., 2018	CCS	II	44	67.8 (Prehabilitation) 69.7 (Controls)	TKA	Education and home-based exercise 12 weeks before surgery	No significant difference in VAS and KOOS occurred at 3 and 6 months between groups. Length of stay was higher for the control group. Of the intervention group, 4 subjects changed their operation decision.
Beaupre et al., 2004	RCT	I	131		TKA	Exercise and education program 4 weeks before surgery	No difference was observed in ROM and strength of the knee, pain and HRQOL

LOE: Level Of Evidence, RCT: Randomized Controlled Trial, WOMAC: Western Ontario McMaster universities Arthritis Index, CS: Case Series, CCS: Case-Control Study, ILAS: Iowa Level of Assistance Scale, THA: total hip arthroplasty, TKA: total knee arthroplasty, VAS: Visual Analogue Scale, ROM: range of motion, TUG: time up and go, 6MWT: 6-minute walking test, 30sCST: 30 s chair stand test, KOOS: Knee injury and Osteoarthritis Outcome Score, HRQOL: Health Related Quality of Life.

**Table 2:** THA.

Study	Type of Study	LOE	N.er of Patients	Mean Age (y)	Type of Scheduled Surgery	Type of Intervention	Outcomes Summary
Hoogeboom et al., 2010	RCT	I	21	76	THA	Preoperative strengthening exercises and tailor-made activity	No difference occurred between study and control groups in length of stay and functional recovery after surgery
Oosting et al., 2012	RCT	I	30	76.9 (study group), 75 (control group)	THA	Supervised walking and functional activities from 6 to 3 weeks preoperatively	Functional improvements were observed with better results in the study group in the postoperative TUG test and 6MWT (6 weeks after surgery)
Wang et al., 2002	RCT	I	28	68.3	THA	Preoperative customized exercise program	Exercise group showed greater stride length and gait speed at 3 weeks after surgery. Gait speed was also greater at 12 and 24 weeks, while 6MWT distance was greater at 12 and 24 weeks

ILAS: Iowa Level of Assistance Scale, THA: total hip arthroplasty, TKA: total knee arthroplasty, VAS: Visual Analogue Scale, ROM: range of motion, TUG: time up and go, 6MWT: 6-minute walking test, 30sCST: 30 s chair stand test, KOOS: Knee injury and Osteoarthritis Outcome Score, HRQOL: Health Related Quality of Life.

**Table 3:** Protocols of intervention.

Study	Main Intervention	Control Group Intervention
Evgeniadis et al., 2008	Trunk and upper extremity elastic band (thera-band) strengthening for the 3 weeks before surgery.	No exercise before surgery, rehabilitation protocol for the 8 weeks after surgery.
Gill et al., 2009	Ingroups of 4 to 6 participants, under physiotherapist instructions: 1 h for 2 times a week for the 6 preoperative weeks. Exercises were completed at a moderate intensity between 12 and 14 on the BRPES. Home exercises were also encouraged 3 times a week. Specific program: “5 to 10 min of forward, sideways, and backward walking; 20 min pedaling a stationary exercise bike; resistance exercises; calf, hamstrings, and quadriceps stretches (2 sets of 30 s).”	Similar program schedule to land based group. Specific program: “walking and active range of movement Exercises; calf, hamstring, and quadriceps stretches (2 sets of 30 s).”
Gstoettner et al., 2011	Preoperative proprioceptive program in the 6 weeks preceding surgery: once a week, 45min training session supervised one-to-one by a physiotherapist. Daily home training instructions were given. Exercises include: slide step forward/backward, step forward/backward, single leg stance and squat.	No preoperative training
Hoogeboom et al., 2010	Supervised training twice a week for the 6 to 3 weeks preceding the index surgery. Four-phases training was administered: “First, patients started with a 5-min walk to warm up. Subsequently, they trained their lower extremity with the leg-press (sets of 10–20) through the full possible range of motion, both concentric and eccentric. Then, patients trained their aerobic capacity on a bicycle ergometer for 20–30 min. Finally, they followed a specific tailor-made training which integrates functional physical activities into the patient’s daily living”	Usual preoperative and postoperative care
Huang et al., 2012	Experienced physiotherapist educated the subjects for a home-based program in a 40 min meeting 2 to 4 weeks before the index surgery. “Exercises included straight leg raising, knee setting, ankle pumping, and hip abduction with resistance”	No activity restrictions in the period before surgery. No specific training or educational, except routine.
Matassi et al., 2012	Patients were instructed for a home. -based program to undertake 5 days a week in the 6 weeks preceding surgery (without the help of a therapist). Exercises included: quadriceps and hamstring stretching, isometric and isotonic quadriceps strengthening, isotonic hamstring contractions, and dynamic stepping.	Regular activities until surgery
Oosting et al., 2012	Supervised 30-min sessions twice a week for the preoperative 6 to 3 weeks. Training was tailored to the patient and his/her environment with focus on walking ability and functional daily activities.	Single supervised session 3 weeks before surgery. The session provided education on postoperative course, walking with crutches and exercises.
Skoffer et al., 2015	Progressive resistance training supervised sessions (60 min each) were undertook 3 times a week in the 4 preoperative weeks and continued the program in the 4 postoperative weeks. After warm-up, the exercise session included leg press, knee extension, knee flexion, hip extension, hip abduction, and hip adduction in strength training machines.	Regular activities until surgery and progressive resistance training program in the postoperative period (4 weeks, as the study group).
VanLeeuwen et al., 2014	Standard strengthening with the adjunction of progressive strength training, including: leg press, step-up, squat, leg extension.	Standard strengthening
Wang et al., 2002	Two supervised sessions and two home-based sessions were scheduled per week, in the 8 weeks before surgery. “All exercise programs were customized to the subject and his or her level of pain, age, and general physical ability”. Supervised sessions included hydrotherapy, stationary bike riding, and resistive training for thigh abduction, thigh flexion and extension, leg flexion and extension, and ankle plantar flexion.	Standard advices on preoperative activities given by the physiotherapist of the hospital.
Williamson et al., 2007	Patients were divided in groups of 6–10 patients and attended 1 h session once a week for 6 weeks before surgery. Exercise circuits were either devised or supervised.	Acupuncture group (II arm) received lower limb acupuncture. Control group (III arm) received an informative leaflet on preoperative exercises and advice
Aytekin et al., 2018	Patients allocated to the intervention group received general education about OA and TKA, with specific home-based exercise program (ankle pumping, knee range of motion, quadriceps isometric, stretching and strengthening).	Regular activities until surgery

BRPES: Borg Rating of Perceived Exertion Scale.

**Table 4:** Cochrane Risk of Bias Assessment Tool.

Study	Sequence Generation	Allocation Concealment	Blinding	Incomplete Data Addressed	Selective Reporting	Other Bias
Evgeniadis, 2008	L	L	U	H	H	H
Gills, 2009	H	H	H	U	L	H
Gstoettner, 2011	L	L	H	U	L	L
Hoogeboom, 2010	L	H	H	U	H	H
Huang, 2012	L	L	H	U	H	H
Matassi, 2012	L	L	H	H	H	H
Oosting, 2012	H	H	H	H	H	H
Skoffer, 2015	L	L	H	U	L	L
VanLeeuwen, 2014	L	L	H	H	H	L
Wang, 2002	H	H	H	U	L	H
Williamson, 2007	L	L	H	U	H	L
Aytekin, 2018	H	H	H	U	L	L

L: Low, H: High, U: Uncertain.
